# Ferroptosis precedes apoptosis to facilitate specific death signalling by fatty acids

**DOI:** 10.1098/rspb.2023.1327

**Published:** 2023-10-25

**Authors:** Valerio Zupo, Maria Costantini, Eliahu D. Aflalo, Tom Levy, Vered Chalifa-Caspi, Olabiyi Obayomi, Mirko Mutalipassi, Nadia Ruocco, Francesca Glaviano, Emanuele Somma, Paola Nieri, Amir Sagi

**Affiliations:** ^1^ Department of Ecosustainable Marine Biotechnology, Stazione Zoologica Anton Dohrn, Villa Dohrn, Ischia, Italy; ^2^ Department of Ecosustainable Marine Biotechnology, Stazione Zoologica Anton Dohrn, Via Ammiraglio Ferdinando Acton, 55, 80133 Napoli, Italy; ^3^ Department of Life Sciences, Ben-Gurion University of the Negev, PO Box 653, Beer-Sheva 8410501, Israel; ^4^ The National Institute for Biotechnology in the Negev, Ben-Gurion University of the Negev, PO Box 653, Beer-Sheva 8410501, Israel; ^5^ Department of Life Sciences, Achva Academic College, Mobile Post, Shikmim 79800, Israel; ^6^ Department of Pharmacy, Pisa University, 56126 Pisa, Italy; ^7^ Interdepartmental Center of Marine Pharmacology, Pisa University, 56126 Pisa, Italy; ^8^ Ilse Katz Institute for Nanoscale Science & Technology, Ben-Gurion University of the Negev, Beer-Sheva 84105, Israel; ^9^ Zuckerberg Institute for Water Research, J. Blaustein Institutes for Desert Research, Ben-Gurion University of the Negev, Midreshet Ben Gurion 84990, Israel; ^10^ Institute for Stem Cell Biology and Regenerative Medicine, Stanford University School of Medicine, Stanford, CA 94305, USA; ^11^ Department of Integrative Marine Ecology, Stazione Zoologica Anton Dohrn, Villa Comunale, 80121 Napoli, Italy; ^12^ Department of Ecosustainable Marine Biotechnology, Stazione Zoologica Anton Dohrn, Calabria Marine Centre, C. da Torre Spaccata, Amendolara, Italy; ^13^ Department of Life Science, University of Trieste, Via L. Giorgieri, 10, 34127 Trieste, Italy; ^14^ Hopkins Marine Station, Institute for Stem Cell Biology and Regenerative Medicine, Stanford University, Pacific Grove, CA 93950, USA; ^15^ NBFC, National Biodiversity Future Center, Piazza Marina 61, 90133 Palermo, Italy

**Keywords:** polyunsaturated fatty acid, diatoms, crustacean, biotechnology, *Hippolyte inermis*, cell death

## Abstract

Cell death is physiologically induced by specific mediators. However, our power to trigger the process in selected cells is quite limited. The protandric shrimp *Hippolyte inermis* offers a possible answer. Here, we analyse a de novo transcriptome of shrimp post-larvae fed on diatoms. The sex ratio of diatom-fed shrimps versus shrimps fed on control diets was dramatically altered, demonstrating the disruption of the androgenic gland, and their transcriptome revealed key modifications in gene expression. A wide transcriptomic analysis, validated by real-time qPCR, revealed that ferroptosis represents the primary factor to re-shape the body of this invertebrate, followed by further apoptotic events, and our findings open biotechnological perspectives for controlling the destiny of selected tissues. Ferroptosis was detected here for the first time in a crustacean. In addition, this is the first demonstration of a noticeable effect prompted by an ingested food, deeply impacting the gene networks of a young metazoan, definitely determining its future physiology and sexual differentiation.

## 1. Introduction

The main pathways of cell death have been outlined, although not yet comprehensively. The attention of scientists has shifted towards their translational aspects, to disentangle the complex regulation and execution of distinct cell-death pathways [1]. Cell death is the ultimate fate for cells that have been damaged, when exposed to lethal signals, but pathways that regulate processes apparently unrelated to cell death, such as autophagy, metabolism, availability of anti-oxidants or organismal ageing, may have a profound influence on the propensity of individual cells to undergo suicide [2]. In fact, death is preceded by a phase at which the cell is subjected to contrasting signals and a decision is finally taken according to an information that is specifically interpreted [3]. In particular, scientists are afflicted by the complexity of human diseases. The absence of convenient model systems limits the power of pathology-oriented approaches and the possibility to understand the dynamic factors triggering the specificity of cell death signals [4], which rule the development and the functions of complex organisms [5]. In this view, we should give priority to simpler approaches, involving suitable invertebrate models, to extrapolate and integrate the multiple factors triggering the production of a death signal addressed [6] towards individual cells and tissues.

Marine invertebrates, such as decapod crustaceans, may represent good models due to the peculiar functions characterizing their sexual determination, which may employ both genotypic sex determination (GSD) and environmental sex determination (ESD) [7]. In GSD, genes are the main factors determining whether given individuals are females or males. In ESD, external stimuli, such as temperature, photoperiod, food quantity and composition [8], and even social factors, control sex determination and, in some crustaceans, these stimuli may assume the role of epigenetic factors.

In the study of sex differentiation—the process that brings about the development of the male or the female phenotype—decapod crustaceans serve as models by virtue of the unique functions of their androgenic gland (AG), which secretes an insulin-like androgenic hormone (IAG) acting as a sexual switch. The IAG-switch [9] gives rise to various sexual systems and also to intersexuality [8,10,11]. Among the species whose sex is determined by ESD, temperature is the determinant factor [12] in most cases, but in amphibians [13] and copepods [14] food may be the determining factor, with the amount of food often serving as a determinant or an inhibitor of differentiation. An important example of food serving as the determining factor for sexual differentiation is provided by the protandric shrimp *Hippolyte inermis* Leach, for which an algal metabolite is the trigger for the development of the female phenotype, as part of coevolutionary processes with *Cocconeis* spp. diatoms. Other examples of foods influencing the sex differentiation are found mainly in insects [15,16].

*H. inermis* is the only known marine invertebrate whose sex determination is strongly influenced by the composition of the food ingested [8,17,18]. The species has evolved a bi-modal reproduction strategy, in which both males and females are produced in the spring, but only males in the autumn, which are to revert their sex about one year later [19]. This unique life cycle, exploiting the advantages of both gonochoristic and protandric strategies, has come about due to the coevolution of diatoms of the genus *Cocconeis* associated with the leaves of *Posidonia oceanica* [8]. In this study, we investigated the genes involved in the sex change of *H. inermis* by feeding young post-larvae with diatoms and then following the gene expression patterns through transcriptomic analyses.

In addition, we studied the effect of the dietary ingestion of the polyunsaturated fatty acid (PUFA) dihomo-γ-linolenic acid (DGLA; 20 : 3n-6), since we posited that a similar lipophilic compound, previously detected in diatom extracts [20], is involved in *H. inermis* sex change by triggering germ-cell ferroptosis, an iron-dependent form of non-apoptotic cell death associated with oxidized polyunsaturated phospholipids [21]. This notion is drawn from a study [22] showing that dietary lipids induce ferroptosis, leading to sterility, in the nematode *Caenorhabditis elegans*. Various authors [23,24] claimed that *C. elegans* cultured in the presence of DGLA became sterile due to the loss of germ cells, sperms and oocytes. Interestingly, exogenous DGLA is sufficient to induce ferroptosis also in human cells, pinpointing this ω-6 PUFA as a conserved metabolic trigger for this kind of cell death [25]. How diet may impact ferroptosis *in vivo* in animal models is less clear, because progresses were hindered by the lack of accessible and easy-to-manipulate animal models exhibiting ferroptosis. Here, we tested the effects of the administration of dietary DGLA, in parallel to the administration of diatoms known to induce sex reversal, to compare the effects of the two treatments, by exploiting the peculiar relationship of a marine shrimp with its diatom food to understand how the ingestion of small fatty acids in early developmental phases relates to the death of undifferentiated cells.

## 2. Methods

### (a) Sample collection and larval development

**Figure 1.**
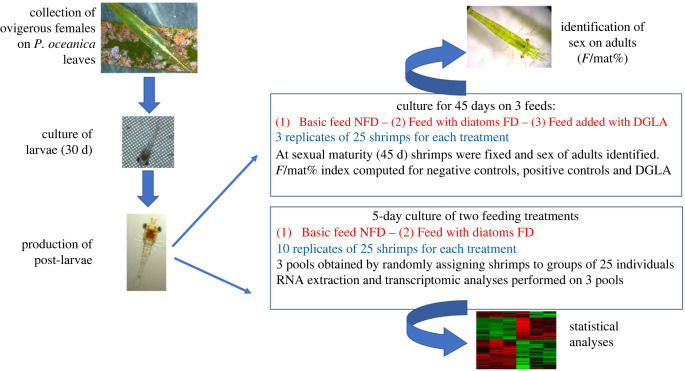
Schematic representation of the experimental plan. The first stage involved collection of ovigerous females from the leaves of *Posidonia oceanica*; hatching and culturing of larvae for 30 days; metamorphosis and production of post-larvae (on the left). From this point, two parallel experiments were conducted: (1) a long-term experiment in which sex was identified at sexual maturity and the ratio of females to mature individuals (F/mat%) was calculated for the treatments FD, NFD and DGLA; (2) a short-term feeding experiment in which pools of shrimps from the treatments FD and NFD were used for RNA extraction, transcriptomic library construction and statistical analysis (represented by a heatmap).

Ovigerous females of *Hippolyte inermis* were collected by using a net dragged by a boat over a *Posidonia oceanica* meadow located off Lacco Ameno d'Ischia (Bay of Naples, Italy). Specimens were collected and macroscopically sorted on the boat, transferred in clean seawater and transported to the laboratory. Identification of shrimps was confirmed under a Leica MZ16 optical macroscope. Further, females were individually transferred to 2 l culture flasks containing 1.5 l of filtered (0.22 µm) seawater. The flasks were maintained in a thermostatic chamber at a constant temperature of 18°C, with 12/12 light/dark cycles. Water was daily changed and sieved through a 0.60 µm plastic net to detect larvae. Hatched larvae were collected, counted, and transferred in pools (larvae from several females) of 80 individuals to 1 l flasks, each containing 800 ml of filtered (0.22 µm) seawater (figure 1, left schemes). Larvae were fed daily on just-hatched *Artemia salina* nauplii (Artemia premium cysts, Super High Group, Ovada, Italy) for about 30 days and regularly inspected to identify the actual time of metamorphosis (while passing through 9–12 zoeal stages). Feeding experiments started on the first day of post-larval growth, when settled post-larvae were individually transferred in replicate pools of 25 individuals in crystallizing dishes containing 400 ml of filtered (0.22 µm) seawater and submitted to various treatments (figure 1) as reported below. According to Italian laws, experiments involving crustacean decapods do not need specific approval by an ethics committee.

### (b) Experimental set-up

In order to obtain sufficient amounts of diatoms to be fed to shrimps, strains (*Cocconeis scutellum* var*. parva*) available in the culture collection of Stazione Zoologica Anton Dohrn were used to prime massive productions in *f/2* medium, further collected, freeze-dried and stored at −20°C until incorporation into the feeds. Three experimental feeds were prepared as follows (figure 1, right scheme): (i) a feed named NFD (no diatom feed) contained equal proportions of dried *A. salina*, dried spirulina and high-protein micro-encapsulates (Microperle, Super High Group, Ovada, Italy) according to Zupo *et al*. [20]; (ii) a second feed named FD (food added with diatoms) was the same basic feed (as NFD) enriched with 1/3 (by weight) of freeze-dried *Cocconeis scutellum* var. *parva*; (iii) in addition, to test the hypothesis that small fatty acids might be responsible for the early death of AG undifferentiated cells, due to ferroptosis [20,22], a third feed was prepared (named DGLA) by adding small amounts of PUFA. In particular, to prepare the DGLA-enriched feed, 45 µl of DGLA (Cayman Chemicals, USA) were dissolved in 1 ml of pure ethanol and added to 450 mg of NFD feed. After a short evaporation (Rotavapor Büchi, Switzerland) the feed was stored at −20°C, until the start of the experiment.

The three feeds were used for a 45-day experimental trial aiming at detecting the effect of diatoms and fatty acids on the sex ratio of shrimps. All feeds were offered at a rate of 5 mg day^−1^ to each replicate pool of 25 shrimp. To this end, settled post-larvae were pooled in three replicates of 25 individuals for each treatment and fed daily on NFD, FD and DGLA diets (figure 1). In the case of DGLA treatment, this procedure assured a dose of 0.004 mg day^−1^ of DGLA per individual shrimp, to adopt a quantity comparable to that reported by Perez *et al*. [22]. The experiment lasted 45 days, up to the reaching of sexual maturity (figure 1). Seawater was daily changed in each crystallizing dish, and cultured post-larvae were monitored and counted under a light microscope (Leica MZ16). Thereafter, adult shrimps were fixed in 70% alcohol and their second pleopods were dissected and analysed under the optical microscope to identify their sex (see electronic supplementary materials for details). The results were expressed as the number of females out of the total number of mature individuals (F/mat%), according to Zupo [8], to compare the effects of three treatments.

### (c) Samples for transcriptomic analyses

In parallel, another experiment was run to detect the molecular mechanisms triggering the selective destruction of the AG [26] leading to sex change, by means of transcriptomic analyses (figure 1). In this case, the early effects of FD and NFD feeds were compared in young (5-day-old) post-larvae. Five-day-old post-larvae were analysed because previous studies [17] demonstrated that the effect of ingested diatoms is prompted within the first 10 days of post-larval growth and any administration of diatoms after this period does not influence the sex of cultured individuals. Consequently, young post-larvae were fixed at halftime of the actual period of activity of diatoms to detect the expression of specific genes in this particular period of their development.

To this end, 10 replicates of 25 just-settled post-larvae were cultured in 400 ml crystallizing dishes and fed on either FD or NFD feeds (totaling 250 individuals for each treatment). On the fifth day, the post-larvae (PL_5_) of each of the two treatments were randomly pooled into groups of 25 individuals held in 2 ml Eppendorf tubes containing 1 ml of RNA Stabilization Reagent (RNAlater, Qiagen, Hilden, Germany) and immediately immersed in liquid nitrogen. The Eppendorf tubes were kept at −80°C until RNA extraction. Excess individuals were released. For each replicate, RNA was extracted, and transcriptomes were obtained and analysed (figure 1, right scheme).

### (d) Sequencing method and RNA-Seq analyses

High-quality, clean and concentrated RNA was extracted—using an EZ-RNA Total RNA Isolation Kit (Biological Industries, Cromwell, CT, USA)—from three biological replicate pools of 25 PL_5_ each, for both FD-fed (feed enriched with diatoms) and NFD-fed (basic feed, without diatoms) post-larvae. The total amount of RNA extracted was estimated by measuring the absorbance at 260 nm and the purity at 260/280 and 260/230 nm ratios, by a Nanodrop (ND-1000 UV-Vis spectrophotometer; NanoDrop Technologies), to exclude the presence of proteins, phenol and other contaminants. RNA integrity was assessed by running about 200 ng of RNA samples in each line of a 6000 Nano LabChip in an Agilent Bioanalyzer (2100 Bioanalyzer, Agilent Technologies, Santa Clara, CA, USA). Samples with high values of the RNA integrity number (RIN), calculated through a comparison of 18S rRNA and 28S rRNA areas, were selected for further processing. About 5 µg of high-quality RNA was dried in an RNAstable kit (Sigma-Aldrich) and used for de novo transcriptome sequencing and assembly (performed by Novogene Company, Hong Kong, China), using an Illumina NovaSeq 6000 platform.

Initial bioinformatic analysis of the sequence reads was carried out using the NeatSeq-Flow platform [27]. Reads were quality trimmed with TrimGalore (https://github.com/FelixKrueger/TrimGalore; accession date January 2021, version 3.0.0). Ribosomal RNA was filtered out as follows: reads were aligned to a database of crustacean rRNA sequences downloaded from the NCBI with BWA MEM [28] using default parameters. Reads that aligned to the rRNA database (12.8–18%) were discarded using SAMtools [29]. A total of 487 865 035 clean reads were retained for further analysis. For constructing the transcriptome assembly, these reads were combined with an additional 1 022 880 897 clean reads from *H. inermis* body samples from different reproductive stages [30]. The transcriptome was de novo assembled using Trinity v. 2.8.4 [31] and then filtered to exclude transcripts with very low expression. To this end, all clean reads were aligned to the transcriptome and only transcripts for which at least one of the experimental groups had at least two replicates with three or more counts per million were retained. The resulting filtered transcriptomes contained 261 562 transcripts (greater than 200 bp) from 119 618 putative genes. The transcriptome was quality assessed using Quast [32] and BUSCO [33] versus the Metazoa_odb9 database. The transcriptome included 98.6% of BUSCO proteins. For transcriptome annotation, the most highly expressed transcript per gene (i.e. the representative transcript) was selected using the filter_low_expr_transcripts.pl script from the Trinity software suite. These representative transcripts were annotated using Trinotate (Trinotate.github.io) by searching Swissprot and PFAM-A, and performing RNAmmer predictions. Best blastx hits and best blastp hits of TransDecoder translated transcripts having an e-value < 1×10^−5^ were reported.

For differential expression analysis, clean reads of the six post-larvae samples (3 FD and 3 NFD) were aligned to the filtered reference transcriptome with Bowtie2 [34] and gene expression was estimated with the software RSEM [35]. Statistical testing for differential expression between FD and NFD was carried out using DESeq2, a method specifically tailored for count data by exploiting negative binomial generalized linear models. Genes were considered differentially expressed (DE) if they had an FDR (false discovery rate) adjusted *p*-value < 0.05 and a linear fold change >1.3 or ≤1.3, where a minus sign denotes downregulation. Hierarchical clustering of the differentially expressed genes, after Z-scoring of their variance-stabilized expression values, was carried out using Partek Genomics Suite. Correspondence analysis was performed using the ‘CA’ R-package, as described by Nenadic & Greenacre [36].

### (e) Generic biological pathway analyses

Transcriptome annotation with Gene Ontology (GO) terms and KEGG Orthology (KO) IDs was extracted from the Trinotate results. In addition, transcript sequences were directly searched against the KEGG database using the KAAS server, by applying the bi-directional best hit (BBH) method. Enrichment analysis of the DE genes against GO Biological Processes was performed using the ClusterProfiler R/Bioconductor package. Mapping of KO IDs to pathways was done using the KEGGREST R/Bioconductor package and overlay of the DE genes on KEGG pathway schemes was done using an in-house script.

### (f) Biological pathway analysis through human orthology

The apoptosis and insulin synthesis pathways were further analysed using orthology to human genes, using our transcriptome of the PL samples (183 044 transcripts, 101 737 genes), and then read alignment, counting and statistical testing were performed as described above. Heatmaps were generated using the PHEATMAP function in R software. An interactive analysis was performed by NetworkAnalyst 3.0 software [37] available at https://www.networkanalyst.ca/, using the STRING interactome of protein–protein interactions [38]. The gene pathways and the physiological processes that they control were identified through orthologous human genes to compute the network analyses. The most notable relations among genes (confidence score cut-off = 900) displaying experimental evidence were highlighted.

### (g) Validation of transcriptome by real time qPCR for genes involved in ferroptosis

Since a ferroptotic cell-death process was described only in a few arthropods [39], and it has been detected here for the first time in a crustacean decapod, further proof of ferroptotic cell death was searched. To provide clear evidence, indicating which cellular players were actually involved in shrimps (in humans, for example, glutathione peroxidase 4 (*GPX4*), ferroptosis suppressor protein 1 (*FSP1*) and acyl-CoA synthetase long-chain family member 4 (*ACSL4*) are known to be involved in this process), we searched and isolated from the shrimp transcriptome the sequences of the genes involved in ferroptosis, and validated their presence and expression levels by means of RT qPCR, in order to provide further evidence of their roles and activity in shrimps (see also Methods in electronic supplementary material). In fact, other experimental approaches would provide doubtful results, given the size of post-larvae and the quick process leading to the disappearance of the androgenic gland. Since the lack of an AG immediately triggers a sex reversal process [26], we also expected that the apoptotic process leading to the destruction of *testis* and *vas deferens* was active in parallel, and consequently such genes as *CIT*, *TSPO* and *DRONC* should be still overexpressed. Consequently, our RT qPCR analyses were aimed at detecting the patterns of activity of those genes, according to the temporal patterns indicated in electronic supplementary material, figure S2.

#### (i) Other statistical analyses

The significance of differences between treatments and controls indicated in figure 1 was tested using a *Z*-test performed with the software tools available in MS.Excel. Plots were produced using Prism ver. 8 for Mac (GraphPad Software, San Diego, CA, USA, www.graphpad.com).

## 3. Results

### (a) Diatoms induce sex reversal in shrimp post-larvae

**Figure 2.**
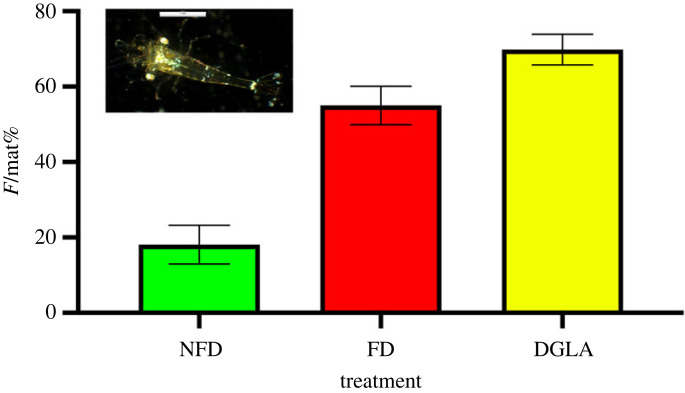
Results of the culture experiments with feeding on three different diets. Inset micro-photo: 5-day-old post-larva (PL_5_) obtained by laboratory larvication and metamorphosis (scale bar, 1 mm). Bar graph: percentage of females out of total mature individuals (F/mat%) obtained following the administration of feed without diatoms (NFD), feed supplemented with diatoms (FD) and feed supplemented with DGLA (DGLA).

Larvae obtained from adult *H. inermis* completed their metamorphosis, settled after 30 days of development and were then used in the feeding experiments. Post-larvae for transcriptomic analyses were grown for five days after metamorphosis (PL_5_ stage) both in the control feed (designated NFD) and in the diatom-enriched feed (designated FD) groups. Both groups exhibited high survival rates (91.9% ± 8.1) and, at the PL_5_ stage, the post-larvae had grown to a length of 3.6 mm (±0.2) (figure 2, inset micro-photo). In addition, post-larvae fed on a diet containing dried diatoms (FD) were cultured for 45 days after settlement (up to a size of about 7 mm); by that time, the percentage of females out of the total of mature individuals (designated F/mat%) reached 55.01 ± 5.10 (*n* = 75) in the FD treatment (figure 2). By contrast, the F/mat% of the NFD treatment (without diatoms) was 18.09 ± 4.94 (*n* = 75) at the age of 45 days. The difference in F/mat% between FD and NFD was statistically significant (*p* < 0.001; *Z*-test). Finally, the F/mat% for DGLA-fed replicates (serving as a positive control for ferroptosis) was 69.84 ± 4.08 (*n* = 75) (figure 2); this ratio differed significantly from that of the NFD treatment (*p* < 0.001; *Z*-test).

### (b) Diatom ingestion influences gene expression

RNA for transcriptomic libraries was extracted from the whole bodies of PL_5_ (both FD and NFD treatment groups). In the entire transcriptome of *H. inermis*, 119 618 genes were identified. Of the identified genes, 8149 were found to be substantially influenced by the ingestion of diatoms (FD versus NFD) at an adjusted FDR *p* < 0.05 and fold change (in linear scale) > 1.3 (electronic supplementary material, table S1).

**Figure 3.**
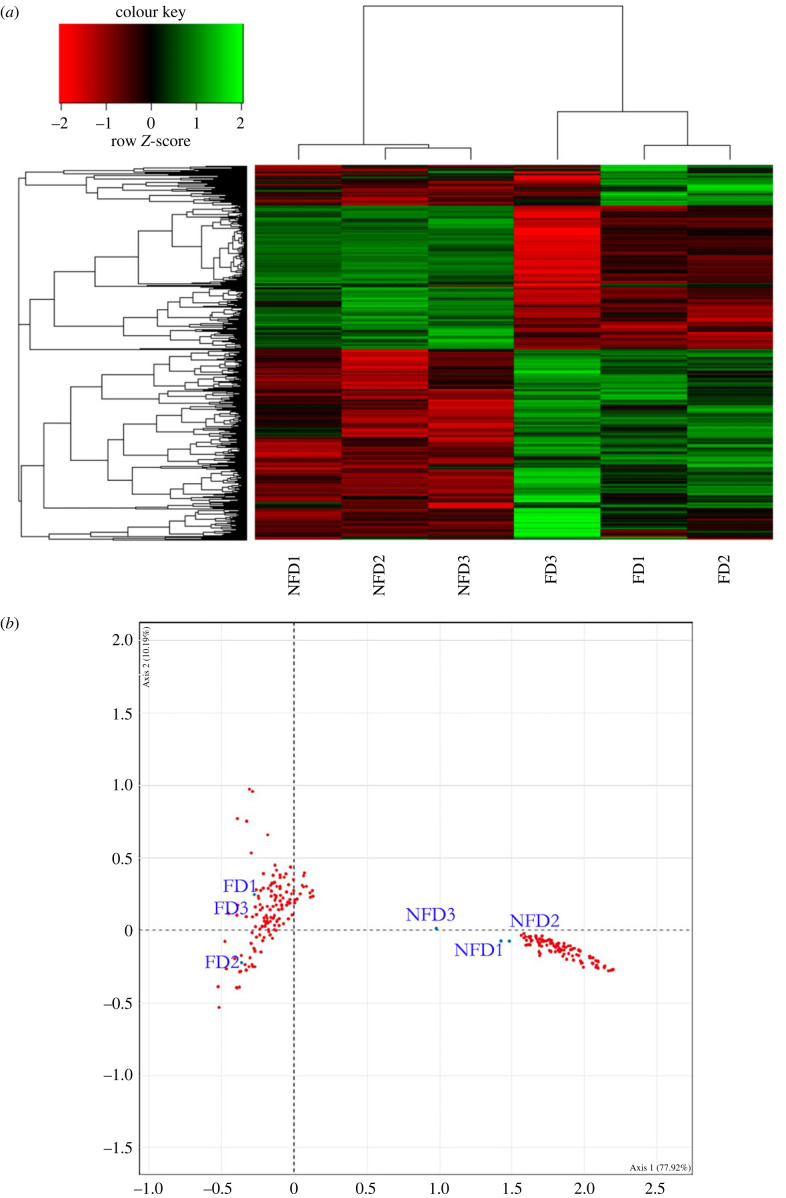
(*a*) Heatmap of differentially expressed genes between shrimps fed on a diet enriched with diatoms (FD1, FD2, FD3) and shrimps fed on a diet without diatoms (NFD1, NFD2, NFD3) adjusted to FDR *p*-value < 0.05 and linear fold change >1.3 or ≤1.3. Genes and samples were clustered based on Pearson's dissimilarity and complete linkage. (*b*) Correspondence analysis performed on a matrix of ‘gene expression versus treatments'. Two clusters (red dots) are visible in the biplot, the FD cluster on the left and the NFD cluster on the right. Blue dots indicate replicates of FD and NFD in the bi-plot factorial plane.

A heatmap of the 8149 differentially expressed genes demonstrated that the replicates fed on diatoms (FD1, FD2, FD3) were significantly different from the replicates fed on diatom-free diet (NFD1, NFD2, NFD3) (figure 3*a*). The analyses of differentially expressed genes provide a picture of the dramatic modifications to the shrimp metabolism, often related to the life and death of cells and tissues. Clear differences in the metabolic pathways were also detected by correspondence analysis, indicating individual genes that mostly contributed to the response to diatom feeding (figure 3*b*). It revealed a core group of genes triggering key metabolic changes and confirmed the noticeable clustering of replicates fed on diatoms (FD) versus those not receiving diatoms in their diet (NFD). The cluster containing NFD shrimps (right side of figure 3*b*) is quite compact in the fourth quadrant. By contrast, the cluster representing the three replicates of shrimps receiving a food containing diatoms (FD; left side of figure 3*b*) is less compact, being distributed in the second and third quadrants, probably due to individually variable metabolic responses induced by diatoms, which impacted the shrimps at various stages of development.

**Figure 4.**
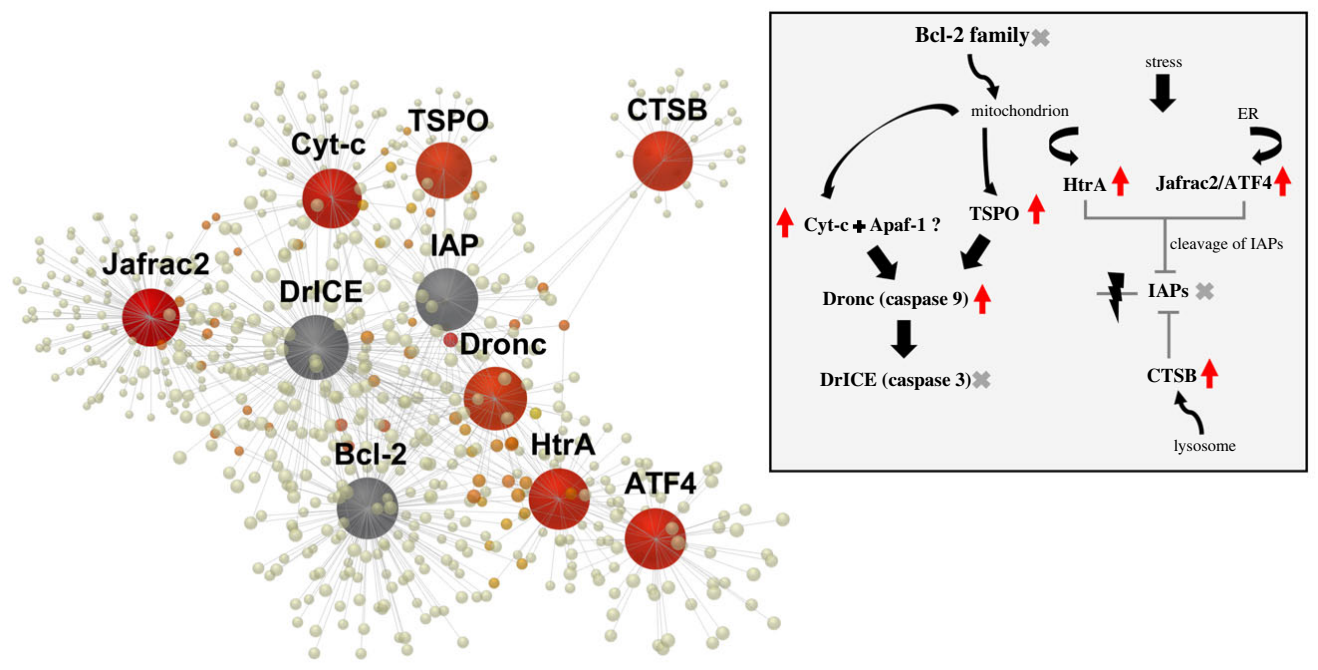
Gene network by interactive analysis, performed by NetworkAnalyst 3.0 software using STRING interactome of protein–protein interactions, including differentially expressed genes for apoptosis, as obtained from KEGG analysis (see electronic supplementary material, figure S4). Grey spheres represent additional connections; red arrows: upregulated genes. Acronyms and names of genes: *Bcl-2*, B-cell lymphoma 2; *Cyt-c*, cytochrome c; *Apaf-1*, apoptotic protease activating factor-1; *TSPO*, translocator protein; *Dronc*, Death regulator Nedd2-like caspase; *DriCE*, Death related ICE-like caspase; *HtrA*, high-temperature requirement A serine peptidase 1; *Jafrac*, Peroxiredoxin 1; *ATF4*, Activating Transcription Factor 4; *IAPs*, Inhibitors of apoptosis proteins; *CTSB*, cathepsin B.

**Figure 5.**
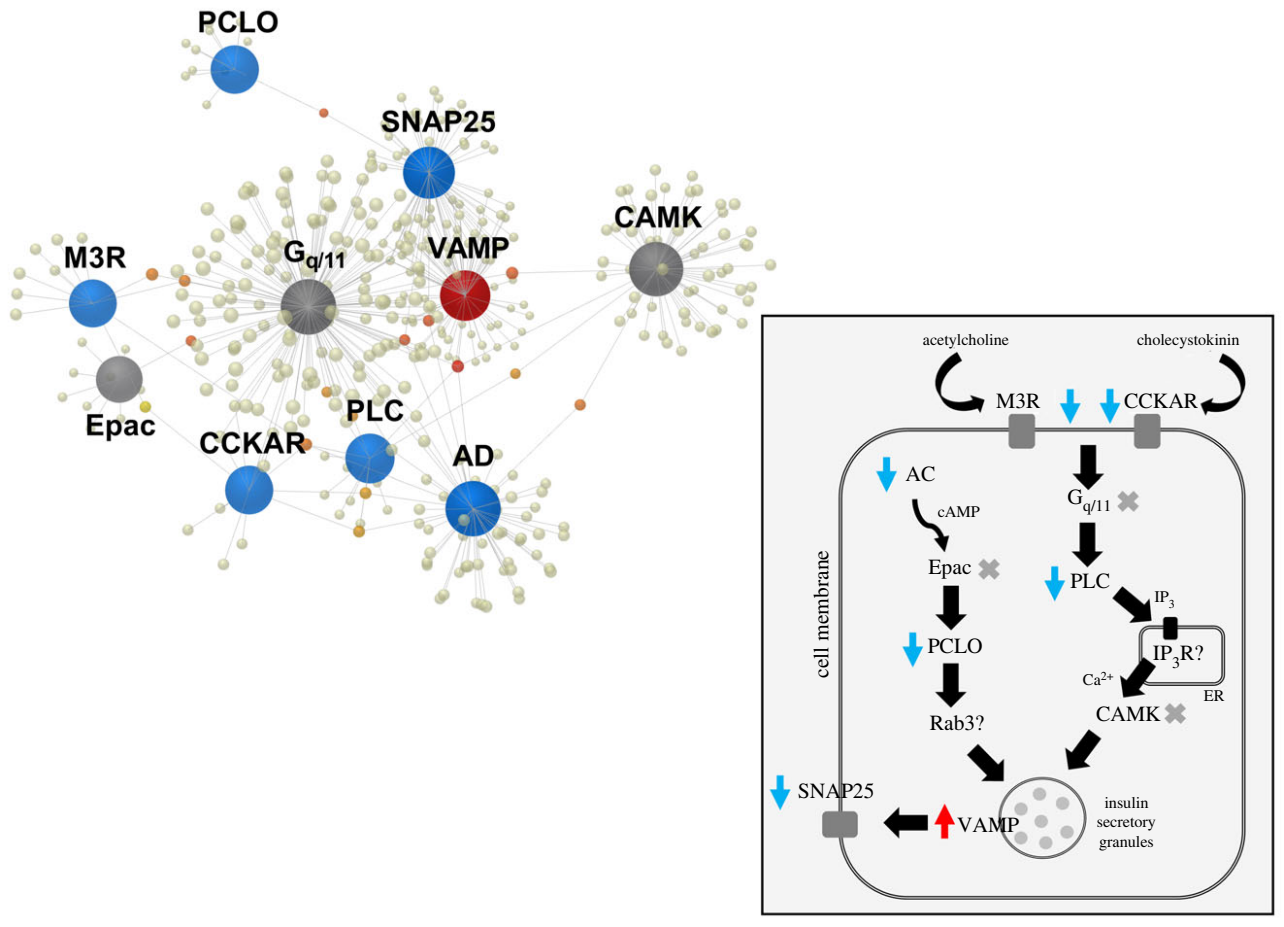
Gene network by interactive analysis, performed by NetworkAnalyst 3.0 software using STRING interactome of protein–protein interactions, including differentially expressed genes involved in the production of insulin-like molecules, as obtained from KEGG analysis (see electronic supplementary material, figure S5). Grey spheres represent additional connections; red arrows: upregulated genes; blue arrows: downregulated genes. Acronyms and names of genes: *MR3*, muscarinic cholinergic receptor; *CCKAR*, Cholecystokinin Type-A Receptor; *AC*, adenylyl cyclase; *Epac*, exchange factor directly activated by cAMP; *PCLO*, Piccolo Presynaptic Cytomatrix Protein; *Rab3*, RAS-Associated Protein; *SNAP25*, synaptosome-associated protein 25; *VAMP*, vesicle-associated membrane protein; *Gq/11*, Gq protein 11; *PLC*, phospholipase C; *IP_3_R*, Inositol 1,4,5-Trisphosphate Receptor Type 3; *CAMK*, Calmodulin-dependent kinases.

Pathway enrichment analysis of the differentially expressed genes versus Gene Ontology (GO Biological Processes revealed that a major portion of the genes was involved in basic cellular processes (electronic supplementary material, table S2) and their differential expression proves the dramatic metabolic changes triggered by the ingestion of diatoms. Of the differentially expressed genes, 1177 genes could be assigned to KEGG database (Kyoto Encyclopedia of Genes and Genomes) pathways (electronic supplementary material, table S3), which include the pathways of ferroptosis, apoptosis and insulin secretion. In particular, six genes involved in apoptosis (figure 4; electronic supplementary material, figure S4) were influenced by the ingestion of diatoms, and they indicate timely transformation processes and a prompt shift leading to tissue re-working at early phases of post-larval life. In parallel, seven genes involved in the insulin secretion pathway (figure 5; electronic supplementary material, figure S5 and electronic supplementary material, table S3), which are part of the metabolism of insulin-like peptides and hormones (such as the insulin-like hormone produced by the AG of male shrimps), were differentially expressed in shrimps fed on *Cocconeis* diatoms. Another important process triggered in diatom-fed shrimps was ferroptosis, which involves six differentially expressed genes (electronic supplementary material, figure S1, table S4). Real time qPCR analyses (see electronic supplementary material, figure S3 and table S4, for values of gene expression variation) provided validation for these results and confirmed the expected patterns of expression of genes promoting ferroptosis (electronic supplementary material, figure S2).

### (c) Diatom ingestion activates remarkable key pathways

Key biological processes affected by the feeding on diatoms were revealed by the overlay of the differentially expressed genes on KEGG pathways, through their assignment to ‘universal’ (non-species-specific) KEGG orthology-based annotation (electronic supplementary material, table S3). To further understand the mechanisms involved in selected pathways, protein–protein interaction networks were computed based on human orthologues of *Hippolyte* genes. In the case of apoptosis, KEGG analysis (electronic supplementary material, figure S4*a*) showed that Apoptosis Inducing Factor 1 (AIF, Hippolyte_Body_TRINITY_DN13768_c1_g2) was significantly upregulated in the FD group, as was the Cytochrome C (*Cyt-c*; 2.2-fold) and the Translocator Protein (*TSPO*; 2.2-fold*).* An increase in expression was also observed for the Peroxiredoxin 4 (*PRDX4,* gene coding for the antioxidant enzyme peroxiredoxin-4; 2.3-fold) and for poly(ADP-ribose) polymerase family member 3 (*PARP3*, gene coding for the protein mono-ADP-ribosyltransferase; 3.0-fold), which plays a key role in the response to the DNA damage surveillance network. In addition, the Basic Transcription Factor 3 *BTF3* was upregulated (3.7), as was the early ecdysone-response Broad-Complex (*BR-C*; 2.9) gene, a key regulator of this cascade encoding for several distinct zinc-finger-containing isoforms. A key gene found downregulated was the Mushroom body large-type kenyon cell-specific protein 1 (*Mblk1*; −3.9 fold), which functions downstream of ecdysone signalling via the ecdysone receptor as an ecdysteroid-regulated protein in morphogenesis during metamorphosis. Finally, for the JACK-STAT (Janus kinases signal transducer) signalling pathway, KEGG analysis showed that pro-epidermal growth factor and cytokine receptors were significantly upregulated in the FD group, while the suppressor of cytokine signalling 7 and serine threonine protein kinase were downregulated (electronic supplementary material, figure S4*b*).

Human orthologues of *Hippolyte* genes showed that several genes involved in the intrinsic mitochondrial apoptotic process were upregulated in response to the ingestion of diatoms (figure 4). In particular, Thioredoxin peroxidase 2 *Jafrac2* (which is rapidly released into the cytosol following the induction of apoptosis), increased (2.4-fold) after the ingestion of diatoms. In addition, the expression of the Activating transcription factor 4 (*ATF4*; 1.7-fold) and of the translocator protein *TSPO* (2.2-fold; present in the outer mitochondrial membrane, in complex with the voltage-dependent anion channel and the adenine nucleotide transporter) was increased. Similarly, an increase in expression was observed for the electron carrier protein *Cyt-c,* leading to altered mitochondrial membrane permeability, and for binding apoptotic protease activating factor-1 (*Apaf-1*) up to the activation of *caspase-9*, which accelerates apoptosis by activating other caspases. Finally, an increase was observed for the Death regulator Nedd2-like caspase (*Dronc*; 5.2-fold) initiator and for cathepsin B (encoded by the *CTSB* gene), which belongs to a family of lysosomal cysteine proteases and contributes to intracellular proteolysis.

In parallel, genes involved in the synthesis of insulin were downregulated, including some key signalling genes, such as the genes encoding the G-protein coupled receptor (*CCKAR*; −3.4-fold), the muscarinic cholinergic receptor (*MR3*; −2.5-fold), adenylyl cyclase (*AC*; 1.7-fold), the protein scaffolding protein (*PCLO*; 3.3-fold), the phospholipase C (*PLC*; −2.4-fold) and the synaptosome-associated protein 25 (*SNAP25*; −1.6-fold), the only exception being members of the synaptobrevin/vesicle-associated membrane protein (*VAMP*; 2.6-fold) family, which were upregulated. The switch-down of the above genes induces a cascade of effects leading to the arrest of the secretion of insulin-like granules (figure 5 and electronic supplementary material, figure S5).

Another pathway related to female differentiation which was impacted by diatom ingestion was the Wnt signalling pathway (KEGG Pathway map04310), which includes the Frizzled receptor, an integral membrane protein involved in multiple signal transduction pathways. In the diatom-fed shrimp, the Wnt signalling pathway exhibited six upregulated genes and two downregulated genes. The upregulated genes included Rbx1 (RING-box protein 1A), SKP1 (S-phase-associated protein 1) and the calcyclin-binding protein (CacyBP)/Siah-1 interacting protein (SIP), with the last two being part of the ubiquitin process, mediating protein degradation by ubiquitination. Additional genes involved in the Wnt pathway were upregulated, such as Rac1 and CK2 (casein kinase II subunit beta). Rac1 is a small signalling G protein belonging to the family of GTPases. Members of this family regulate a diverse array of cellular events, including the control of GLUT4 translocation, cell growth, cytoskeletal reorganization, antimicrobial cytotoxicity and the activation of protein kinases. CK2 is the regulatory subunit of casein kinase II, a kinase complex that phosphorylates several substrates.

## 4. Discussion

The ingestion of diatoms by young post-larvae of *H. inermis* produces a sequence of physiologic events*,* leading to a sexual shift from male to female [8]. The transformation towards femaleness, which is mediated by the IAG-switch [40], was also recently demonstrated in the protandric shrimp *Pandalus platyceros* [40]. However, *H. inermis* represents a unique case, because its sex shift, in spring, is triggered in post-larvae [17] by the ingestion of diatoms and also because the sex shift is completed in the frame of a single moult cycle [18,41], without passing through a distinguishable transitional ovotestis-bearing stage [42]. This rapid transition from a male to a female phenotype is associated with the transcriptomic changes described above. The elucidation of the diatom active compound that is responsible for the sex change is still incomplete [20], but it is believed to be an unidentified highly lipophilic 21-carbon molecule [43]. Here, for the first time, we conducted a transcriptomic investigation of sexually transforming shrimp juveniles, which provides evidence of the early effect of diatoms as a food source [44]. All replicates of the FD-fed shrimps exhibited similar patterns of physiologic variations, which were different from those of the NFD-fed shrimps. Moreover, most NFD-fed shrimps further developed and matured after 45 days as males, while most FD-fed shrimps developed as females.

### (a) Ferroptosis initiates cell death processes

Various cell-death processes could trigger the destruction of the AG, as the first event priming further cascades of modifications. Our transcriptomic analyses demonstrated that genes involved in ferroptosis, an iron-dependent form of non-apoptotic cell death associated with oxidized polyunsaturated phospholipids [20], were consistently upregulated in FD-fed shrimps. According to known mechanisms in human cells [21,45], a ferroptotic process should be primed by the inactivation of the gene *FSP1* (suppressor of the ferroptosis), followed by a short-time increase of the key gene *ACSL4*, which in its turn activates the genes serine acetyltransferase (*SAT1*), *GPX4*, gamma-glutamylcysteine synthetase (*GSHI*) and Six-Transmembrane Epithelial Antigen of Prostate 3 (*STEA3*). Since the process must be started by the ingestion of diatoms, immediately after the larval settlement, we expected that (electronic supplementary material, figure S2), at the fifth day, when the process is in advanced progress in most post-larvae, *FSP1* should be not activated (because it would suppress the process), as well as *ACSL4* (because it completed its activation role), while *SAT1*, *GPX4*, *GSHI* and *STEA3* could be still activated in FD shrimps, up to the complete destruction of the AG tissues. Our hypotheses were fully confirmed by the RT qPCR validation.

Ferroptosis is a highly conserved mechanism along evolution, affecting cells of nematodes and crustaceans and even human cancer cells [45]. It is characterized by three conserved hallmarks: the presence of oxidized PUFAs and redox-active iron and compromised lipid peroxide repair [21]. The process may be triggered by various fatty acids [22]. For example, dietary administration of DGLA was found to trigger germ-cell ferroptosis and sterility in *Caenorhabditis elegans* [46]. In the current study, after demonstrating that genes involved in ferroptosis were differentially expressed specifically in FD-fed shrimps, we performed an additional bioassay in which we added DGLA (a 20-carbon-chain fatty acid, unsaturated at positions 8, 11 and 14) to the diet of shrimps with the aim to further explore the hypothesis that dietary lipids could trigger the phenotypic sex shift in *H. inermis* [27]. Dietary DGLA is a potent inducer of germ-cell death in *Caenorhabditis elegans* [23,47]. Indeed, DGLA at a low concentration produced similar phenotypic effect to that of the diatoms (figure 2). Thus, it is likely that two different types of cell death occurred sequentially in *H. inermis*, leading to the sex shift. We posit that the lipophilic compound carried by the benthic diatoms selectively destroys the immature AG of the shrimps by a ferroptotic process and that, consequently, a cascade of apoptotic processes destroying all male characters is triggered by the lack of IAG hormone, leading to the re-programming of the shrimp body towards femaleness [48]. It is thus suggested that the ferroptotic process is quite specific, affecting the cells of the AG, when shrimps are exposed to the lipophilic trigger during early post-larval development. The similarity between the effects of the diatoms and DGLA in the diet suggests that similar lipids (small molecules containing 20–21 carbons) are likely to induce the death of the AG germ cells in *H. inermis*. It should be noted, however, that the ability of exogenous DGLA to induce ferroptosis was not cell-type restricted [22], whereas the putative compound present in *Cocconeis* spp. was demonstrated to be specific to the germ cells of the AG. The identification of this compound (which is currently underway) is of importance for understanding the nature of its specificity and the coevolutive processes leading to the unique plant–animal communications set between *H. inermis* and its diatom food.

The results obtained permit one to draw the activity of individual genes and sketch the whole process leading to the sex reversal after the ingestion of diatoms, which is in agreement with our initial hypotheses. We hypothesized that in the cells of the immature AG of *H. inermis*, as in human cells, transferrin (*TF*) and transferrin receptor *(TfR*) cooperate to transport Fe^3+^ from the extracellular space to the intracellular space. Ferritin and its related genes, ferritin light chain (*FTL*) and heavy chain 1 (*FTH1*), were expected to modulate the storage of iron ions. In human cells, the Nuclear E2-related factor 2 (Nrf2) and the heat-shock protein B1 reduce the intracellular concentration of iron by suppressing the expression of Transferrin Receptor 1 (TfR1) and limiting reacting oxygen species production, to inhibit ferroptosis [49]. The results of real time qPCR confirm that *FSP1* was no more expressed at the fifth day in post-larvae fed on diatoms (electronic supplementary material, figure S3 and table S4). In the meanwhile, the *ACSL4* gene was expected to be expressed only in the first days after diatom administration, in order to regulate the expression of *SAT* and *GPX4* genes (see also electronic supplementary material, figure S3), which in turn are activated by *GSH1* and *STEAP3* genes. In fact, they were all upregulated at the fifth day, as expected, to complete the ferroptotic process. When shrimps were collected, five days after the start of the ferroptotic process, also apoptosis was activated, because the lack of IAG activity immediately promotes the destruction of *testes* and *vas deferens* [8,9]. In fact, the B-cell lymphoma 2 (*Bcl-2*) apoptosis regulator gene and all the genes involved in this process were upregulated.

Our findings, obtained through a de novo transcriptomic analysis and validated through RT qPCR, may lead to a paradigm shift in our view of ferroptosis signalling, because the premise that the pro-ferroptotic effects of DGLA are uniquely displayed by *C. elegans* [20] should be revised, based on the results of the present study. DGLA-induced ferroptosis is specifically directed towards the germ cells of young organisms and this process is similar to the diatom-induced cell death of germ cells in *H. inermis*. This similitude is of paramount scientific interest, because it indicates powerful ways to control the development of given tissues. Possibly, the germline of the AG may be deficient in antioxidant defences (or enriched in pro-oxidant enzymes), thereby rendering these cells hypersensitive to the accumulation of lipid peroxides, compared with somatic cells. It is also likely that ferroptotic death could be the key process inducing sex reversal in adult decapods, in the absence of diatoms, since it has been demonstrated that the ageing process itself may lead to a loss of control over iron homeostasis or other processes that normally prompt ferroptosis in somatic tissues [50].

### (b) The sex differentiation is completed

The fast protandric shift of *H. inermis* upon the ingestion of diatoms is mediated by the death of the AG germ cells [42,51], followed by or in parallel to the apoptosis of testes and *vas deferens* cells [8,42]. Indeed, our transcriptomic analyses showed elevated apoptotic processes in FD-fed shrimps through the *Dronc* pathway. Since the destruction of the AG causes the cessation of IAG secretion, ‘shutting off’ the IAG-switch [9] and directing the differentiation towards femaleness, it is suggested that diatom-triggered ferroptosis of the AG cells is the initial event in the above-mentioned protandric cascade of events.

Pro-apoptotic genes, such as *Cyt-c*, were noticeably upregulated upon the ingestion of diatoms (FDR adjusted *p*-value 0.0084, linear fold change 2.81) through activating caspases [52,53]. Consequently, the suggested initial destruction of the AG tissue by ferroptosis is followed promptly by a cascade of pro-apoptotic messages, leading to modification of the male primary characters (testes, *vas deferens*, gonopores) and the re-modelling of secondary sexual characters. Consequently, a single death signal targeting a small gland (the AG) leads to wide changes in signalling pathways, inducing the re-design of several budding organs. The production of IAG [54] is among the main processes interrupted by the ingestion of diatoms, since it is known that insulin-like peptides (IAG) determine the development and maintenance of the male sex condition in decapod crustaceans [4,55]. Genes involved in insulin-like hormone production were upregulated in NFD-fed shrimps, while a marked downregulation characterized the FD-fed shrimps, starting from the membrane mediator genes *CCKAR* and *M3R*, activating a cascade of gene downregulation up to the final production of hormone granules. Downstream elements involved in the signalling cascade were identified here for the first time (electronic supplementary material, figure S5), indicating that processes leading to the secretion of IAG start as early as a few days after post-larvae settlement. The long list of receptor-encoding genes and signalling encoding genes influenced by the ingestion of diatoms emphasizes the complexity of sexual differentiation in decapod crustaceans and the maintenance that takes place in the 5-day-old post-larvae (PL_5_).

### (c) Cascades of physiological modifications are complex

**Figure 6.**
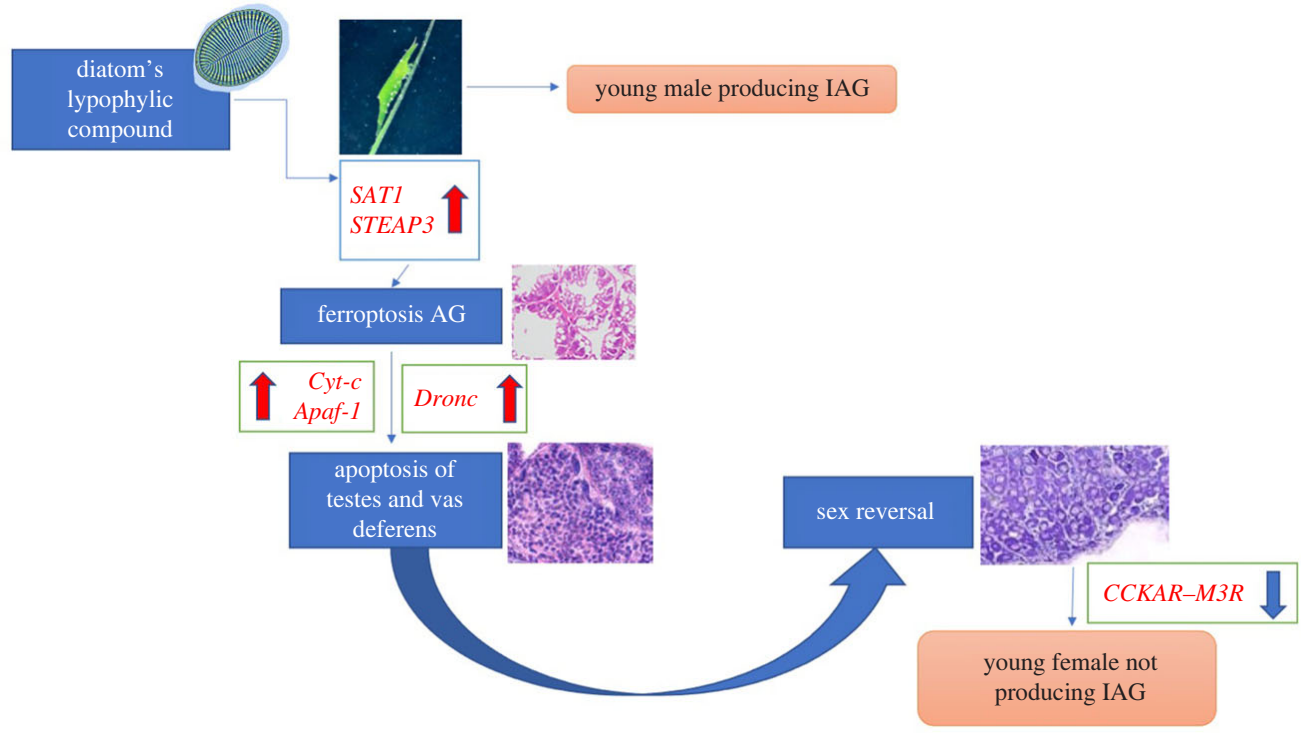
Representation of the main processes derived from the analysis of the transcriptome of *H. inermis* upon the ingestion of *Cocconeis* spp. The lipophilic compound present in the diatoms (as modelled by DGLA) activates *SAT1* (Spermidine/Spermine N1-Acetyltransferase 1) and *STEAP3* (Six-transmembrane epithelial antigen of prostate 3 metalloreductase) genes, inducing a process of ferroptosis specifically in the tissues of the rudimentary androgenic gland of 5-day-old post-larvae. Following the destruction of the androgenic gland, male primary and secondary sex characters, including the testes and *vasa deferentia*, undergo a process of apoptosis, starting with the upregulation of the electron carriers *Cyt-c* and *Apaf-1*, which in turn triggers the activation of the *Dronc* initiator, leading to a complete sex shift. The production of the IAG hormone is stopped through the downregulation of *CCKAR* and *M3R*. According to this scheme, following the apoptosis of the male reproductive system (primed by ferroptosis specifically destroying the AG tissues), the sex reversal occurs, and the female reproductive system develops.

The dramatic physiological alterations detected after the feeding of diatoms to *H. inermis* post-larvae may be summarized into a few logically connected steps, as proposed in figure 6. Initially, the male post-larvae, still not completely developed, produce IAG. The ingestion of diatoms induces ferroptotic death [22] of the AG germ cells [41] due to the action of a lipophilic compound [20]. The consequent lack of IAG causes apoptotic destruction of primary and secondary male characters, such as the testis and *vasa deferentia* [43]. The molecular signalling initiated by the lipophilic compound is accompanied by an array of physiological cascades, which is reflected in the long list of signalling molecules differentially expressed in FD-fed shrimps as compared to NFD-fed shrimps. Similar processes are mimicked by the administration of DGLA. This process leads to the sex change and, consequently, the newly developed ovary starts the production of a different array of hormones.

### (d) Cell death: biotechnological implications

As indicated above, several genes related to *H. inermis* cellular metabolism exhibited a changed level of expression upon ingestion of the diatoms. The exploitation of ferroptosis to manipulate the sex of shrimps and prawns in order to obtain all-male or all-female populations [44] leads to aquaculture applications. For such a biotechnology to be brought to fruition, a detailed elucidation of the ferroptotic process primed by the administration of anti-oxidants and lipid peroxides will be necessary. In addition to the aquacultural potential of our findings, this study has far wider implications for the manipulation of cellular processes. For example, combining exogenous fatty acids with small-molecule inducers of ferroptosis may constitute an effective means of inducing ferroptosis *in vivo* in selected cells. Previous investigations [44,49] have indeed demonstrated that crude extracts of *Cocconeis* spp. selectively trigger cell death in various types of cancer cells *in vitro*. Perhaps more importantly, the results of this investigation indicate the possibility that the failure to combat lipid synthesis and cellular oxidative stress, and possibly the failure to prevent excessive ferroptotic cell death after exposure to dietary PUFAs, might open the way to the treatment of various diseases [8]. In particular, a full comprehension of the ferroptotic cascade may lead to the development of anti-cancer drugs. The above results establish *H. inermis* as a powerful and genetically tractable animal model for apoptosis and ferroptosis [47]. The cascade of apoptotic processes following the destruction of the AG is known from previous studies [43,44], but the discovery of such an intense metabolic modification in 5-day-old shrimps was quite unexpected. Here we demonstrated that a specific food ingested in very early stages of development may produce decisive changes for the life of a metazoan.

## Data Availability

Most source data are provided with this paper, in the main text and electronic supplementary material. The transcriptome sequences are stored in a large database, physically located at the Ben Gurion Informatic facilities, and available on request from the authors. Supplementary material is available online [56].
